# Supplementary figures and images for: A rescue assay for genetic diagnosis of oculocutaneous albinism using melanocytic MNT1 knock-out cells
Source: Front Genet. 2026 Jun 19;17:1821461. doi: 10.3389/fgene.2026.1821461 (PMC13327656; doi:10.3389/fgene.2026.1821461)

**Figure S1**

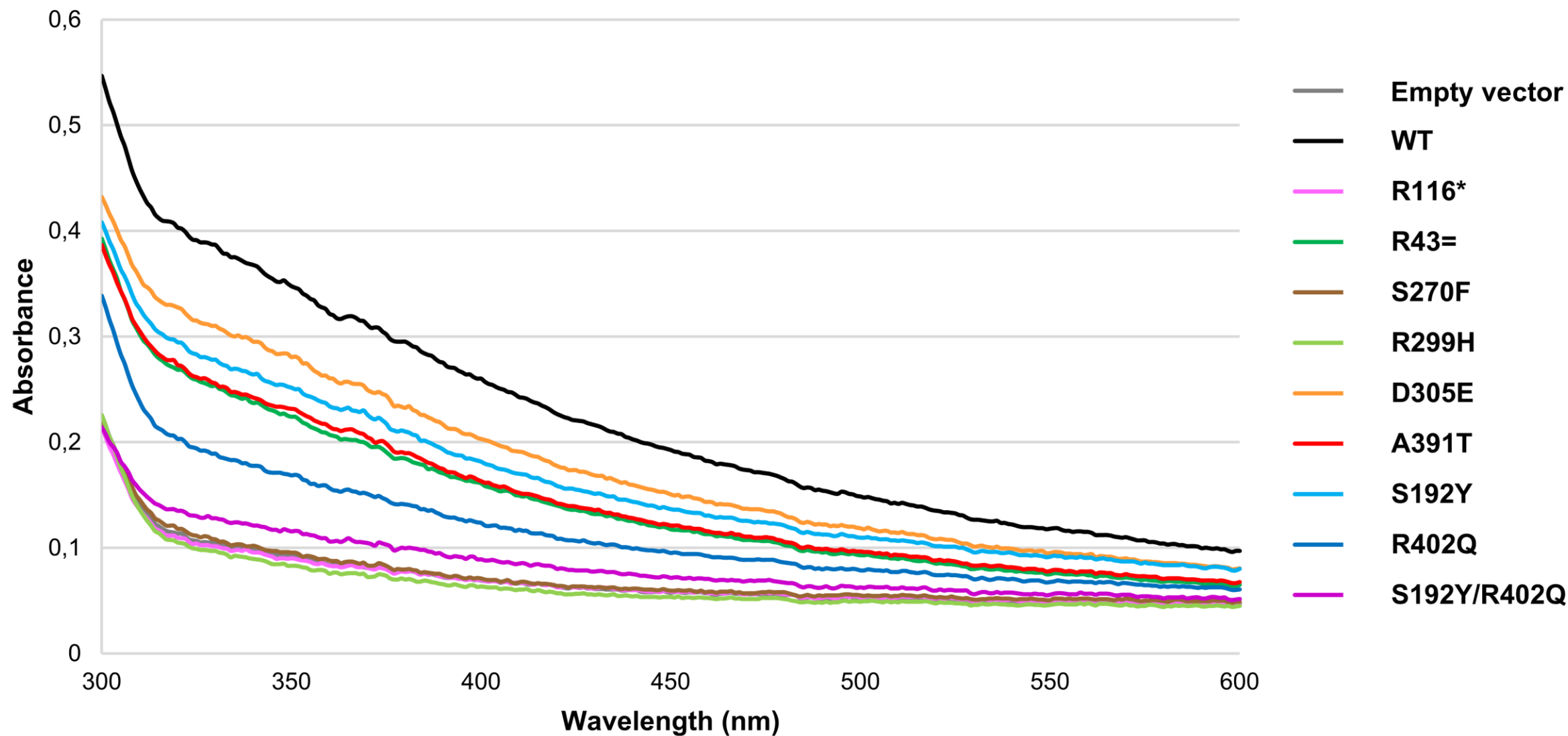

**Figure S2**

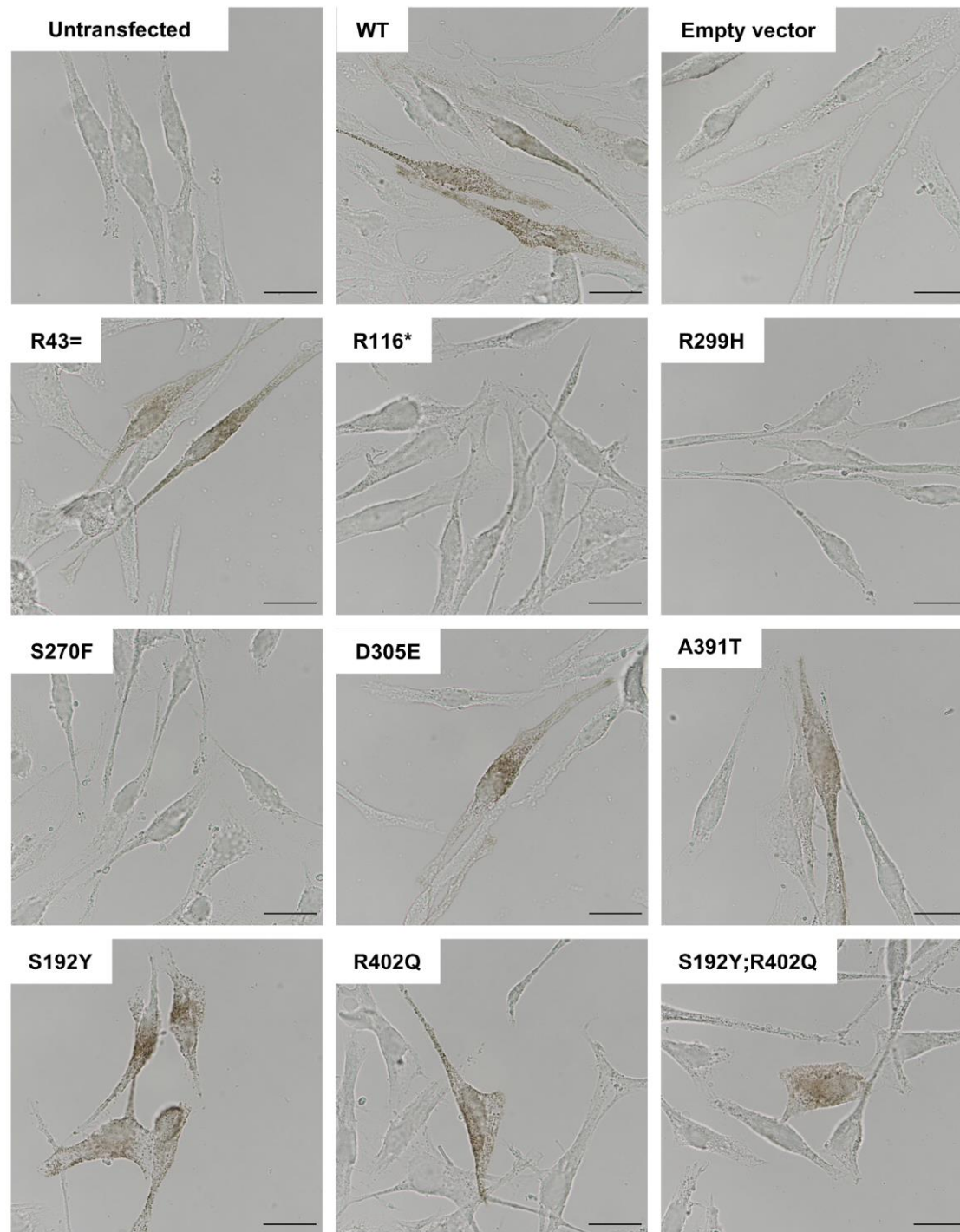

**Figure S3**

**(a)**

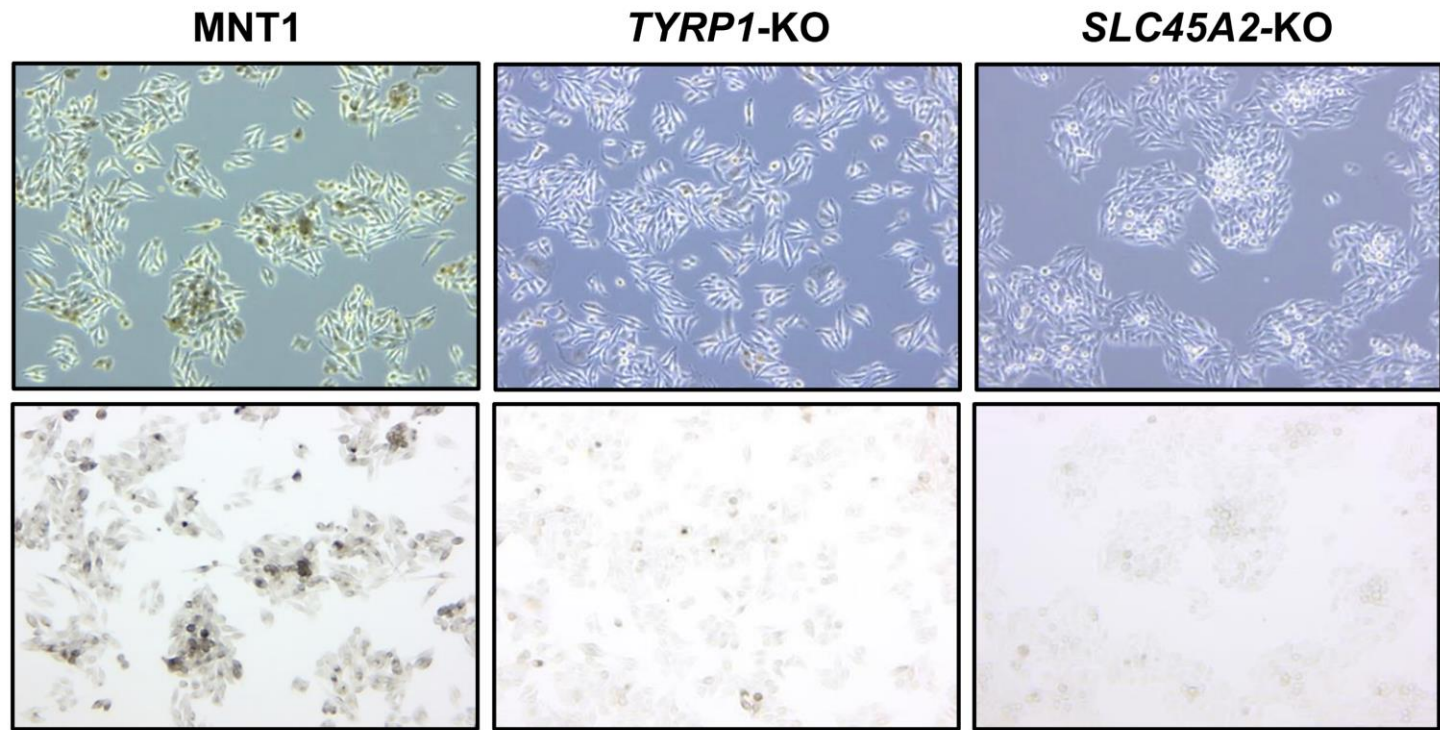

**(b)**

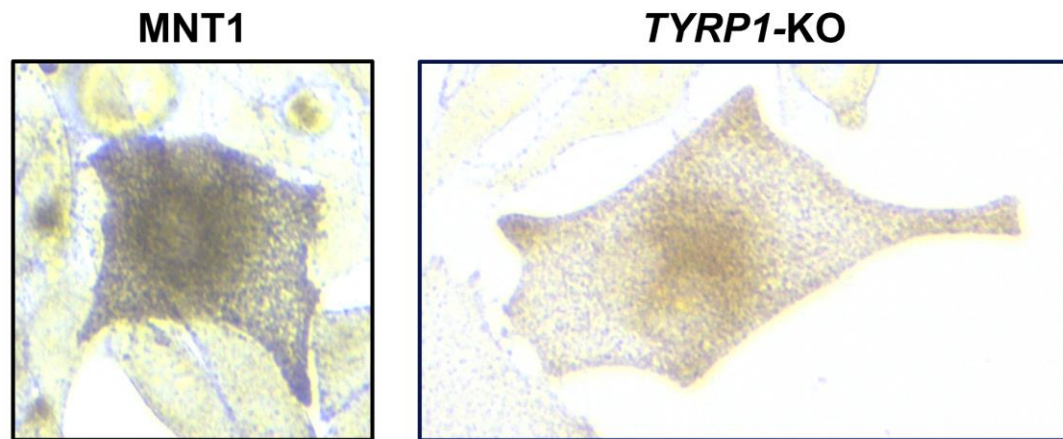

Supplement: Supplementary file 2 [file DataSheet1.pdf]
