# Supplementary material for: A rescue assay for genetic diagnosis of oculocutaneous albinism using melanocytic MNT1 knock-out cells
Source: Front Genet. 2026 Jun 19;17:1821461. doi: 10.3389/fgene.2026.1821461 (PMC13327656; doi:10.3389/fgene.2026.1821461)
Supplement: Supplementary file 3 [file Supplementaryfile1.pptx]

## Slide 1
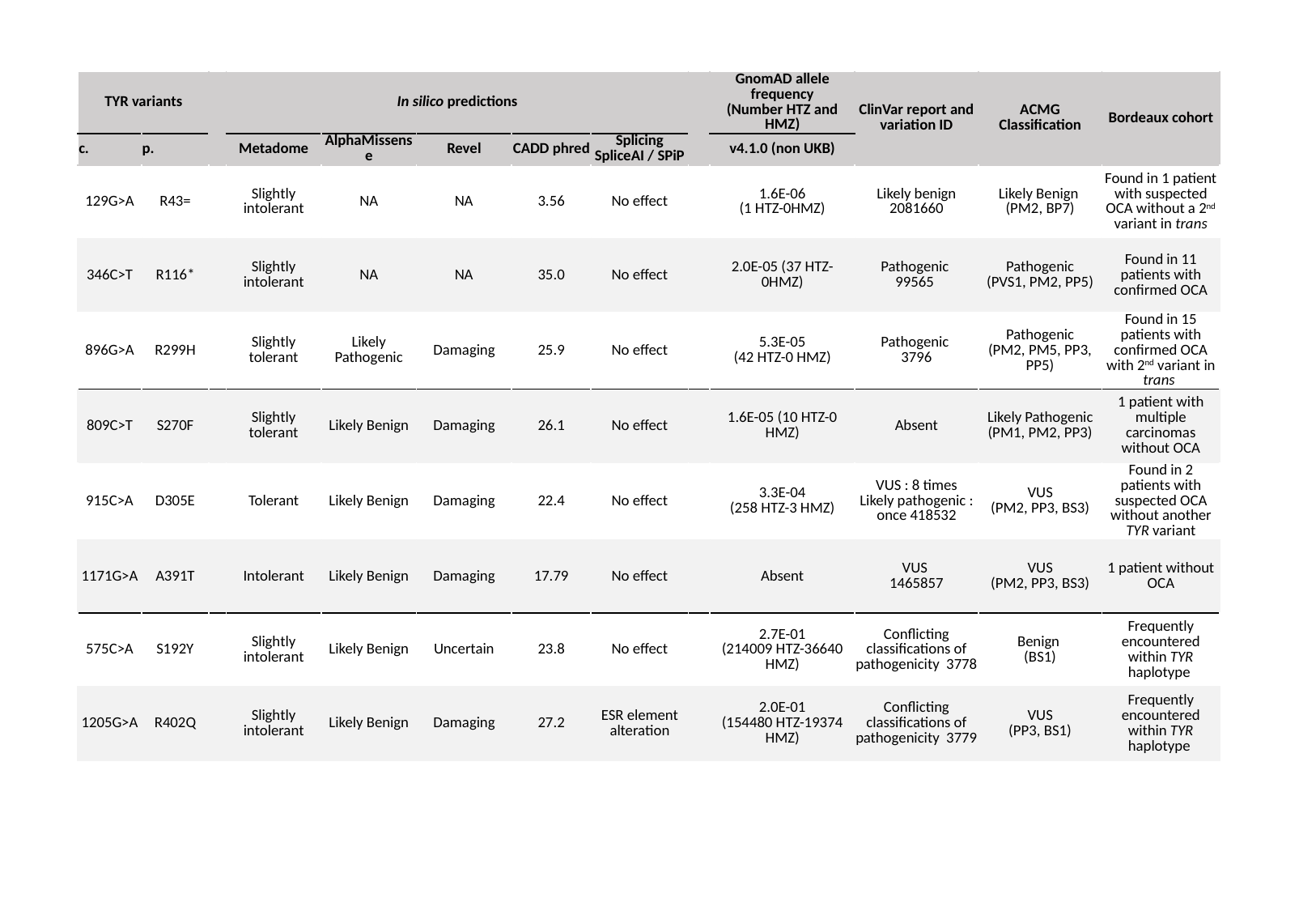

| TYR variants | | | In silico predictions | | | | | | GnomAD allele frequency (Number HTZ and HMZ) | ClinVar report and variation ID | ACMG Classification | Bordeaux cohort |
| --- | --- | --- | --- | --- | --- | --- | --- | --- | --- | --- | --- | --- |
| c. | p. | | Metadome | AlphaMissense | Revel | CADD phred | Splicing SpliceAI / SPiP | | v4.1.0 (non UKB) | | | |
| 129G>A | R43= | | Slightly intolerant | NA | NA | 3.56 | No effect | | 1.6E-06 (1 HTZ-0HMZ) | Likely benign 2081660 | Likely Benign (PM2, BP7) | Found in 1 patient with suspected OCA without a 2nd variant in trans |
| 346C>T | R116\* | | Slightly intolerant | NA | NA | 35.0 | No effect | | 2.0E-05 (37 HTZ-0HMZ) | Pathogenic 99565 | Pathogenic (PVS1, PM2, PP5) | Found in 11 patients with confirmed OCA |
| 896G>A | R299H | | Slightly tolerant | Likely Pathogenic | Damaging | 25.9 | No effect | | 5.3E-05 (42 HTZ-0 HMZ) | Pathogenic 3796 | Pathogenic (PM2, PM5, PP3, PP5) | Found in 15 patients with confirmed OCA with 2nd variant in trans |
| 809C>T | S270F | | Slightly tolerant | Likely Benign | Damaging | 26.1 | No effect | | 1.6E-05 (10 HTZ-0 HMZ) | Absent | Likely Pathogenic (PM1, PM2, PP3) | 1 patient with multiple carcinomas without OCA |
| 915C>A | D305E | | Tolerant | Likely Benign | Damaging | 22.4 | No effect | | 3.3E-04 (258 HTZ-3 HMZ) | VUS : 8 times Likely pathogenic : once 418532 | VUS (PM2, PP3, BS3) | Found in 2 patients with suspected OCA without another TYR variant |
| 1171G>A | A391T | | Intolerant | Likely Benign | Damaging | 17.79 | No effect | | Absent | VUS 1465857 | VUS (PM2, PP3, BS3) | 1 patient without OCA |
| 575C>A | S192Y | | Slightly intolerant | Likely Benign | Uncertain | 23.8 | No effect | | 2.7E-01 (214009 HTZ-36640 HMZ) | Conflicting classifications of pathogenicity 3778 | Benign (BS1) | Frequently encountered within TYR haplotype |
| 1205G>A | R402Q | | Slightly intolerant | Likely Benign | Damaging | 27.2 | ESR element alteration | | 2.0E-01 (154480 HTZ-19374 HMZ) | Conflicting classifications of pathogenicity 3779 | VUS (PP3, BS1) | Frequently encountered within TYR haplotype |
